# LncRNA PVT1 Promotes Cell Proliferation, Invasion, and Migration and Inhibits Cell Apoptosis by Phosphorylating YAP

**DOI:** 10.1155/2022/5332129

**Published:** 2022-05-26

**Authors:** Kaiyue Ji, Qi Zhang, Wen Song, Tao Mao, Jing Guo, Congcong Min, Cuiping Zhang, Zibin Tian, Xiaoyu Li

**Affiliations:** Department of Gastroenterology, Affiliated Hospital of Qingdao University, Qingdao University, Qingdao, China

## Abstract

Gastric cancer (GC) as a serious global health problem is a threat to human longevity. Plasmacytoma variant translocation 1 (PVT1) participates in the formation and progression of various cancers, including GC. The aim of this study is to investigate the mechanism underlying the functions of PVT1 and explore a novel target for the diagnosis and treatment of GC. Analysis of the TCGA dataset using the R software identified that the lncRNA PVT1 was greatly upregulated in GC tissues. Twenty pairs of GC and adjacent normal tissues were acquired from patients with GC, and the expression of PVT1 was evaluated using RT-qPCR. Furthermore, PVT1 expression was knocked down in GC cells using siRNA, and the GC cells were divided into control, negative control (NC), and siRNA groups. Cell proliferation ability was analyzed using Cell Counting Kit-8 (CCK8) and colony formation assays, whereas cell migration and invasion ability were investigated through wound healing and Transwell assays. Moreover, Western blotting was used to analyze the expression of Yes-associated protein (YAP) and epithelial-to-mesenchymal transition (EMT) proteins. We also found that PVT1 and YAP expressions were upregulated in the GC tissues compared with those in the adjacent nontumor tissues. Knockdown of PVT1 was found to inhibit the proliferation, invasion, and migration and promote apoptosis of GC cells. Furthermore, knockdown of PVT1 downregulated YAP and promoted phosphorylation of YAP, suggesting that PVT1 exerts actions on GC cells by targeting YAP and inhibits cell apoptosis in vitro. The EMT process was also inhibited by the knockdown of PVT1. In summary, lncRNA PVT1 facilitated cell proliferation, invasion, and migration and suppressed cell apoptosis by targeting YAP. This study suggests that the expressions of PVT1 and YAP could be used for the early detection of GC and the occurrence and development of GC could be inhibited by interfering the interaction of PVT1 and YAP, which will provide new insights for the diagnosis, treatment, and prognosis of GC.

## 1. Introduction

Gastric cancer (GC) as a severe global health problem is one of the major threats to global health and human longevity [1]. As the fifth most prevalent type of malignant tumor, GC has become the third leading cause of cancer-related death all over the world [2]. Owing to the advancement of endoscopic diagnosis and treatment technology, many early-stage gastric cancers can be detected and resected using endoscopic submucosal dissection. However, early diagnosis may not always be possible because of the insidious symptoms in the early stage of the disease [3]. Hence, most patients are diagnosed with advanced gastric cancer with metastasis when detected [4]. Despite the advances in treatment strategies, the overall prognosis of GC is poor due to tumor recurrence and metastasis [5]. Hence, it is crucial to find out the molecular biological mechanism underlying the progression of the disease and explore therapeutic targets for early diagnosis and treatment of GC.

Long noncoding RNAs (lncRNAs) are transcripts over 200 nucleotides in length with no or low protein-coding potential [6–8]. Abnormalities in lncRNAs may lead to the formation of tumors by promoting cell proliferation, invasion, and metastasis and inhibiting apoptotic mechanisms of cells [9]. Thus, detecting changes in lncRNAs in the early stages of tumor formation and development is essential for the treatment of tumors. Some lncRNAs have been found to interact with proteins. Thus, elucidating the regulatory mechanisms between lncRNA and protein expression may create novel insights for the therapeutics of various tumors and other diseases [10].

Plasmacytoma variant translocation 1 (PVT1), located at 8q24.21, has a large locus of over 30 kb in length [11]. The locus provides a special site of genetic abnormalities, including amplification, translocation, and many risk loci in cancers or various diseases [12]. It has been clarified that the PVT1 expression in many malignant tumor cell lines and tumor tissues is higher than that in normal tissues including GC and colorectal cancer [13, 14]. Furthermore, some studies have suggested that PVT1 is a mediator of cancer progression [15].

The Hippo pathway, frequently downregulated in human cancers, has an important influence on regulating cell plasticity, organ growth, and tissue homeostasis [16]. Yes-associated protein (YAP) as the major effector of the Hippo pathway has the ability to inhibit it [17]. The transcriptional coactivator YAP exerts a vital effect on cell proliferation, differentiation, and apoptosis [18]. The transcriptional ability of YAP relies on the position of YAP in cells. YAP enhances the transcription of a series of downstream genes, together with transcriptional activators, and exerts a pleiotropic effect in tumor growth and inhibition of apoptosis when it enters the nucleus. When YAP binds to phosphorylated ser127 and accumulates in the cytoplasm, it prevents YAP from entering the nucleus and causes loss of its function as a transcriptional coactivator [19].

The epithelial-to-mesenchymal transition (EMT) is an important biological process that naturally occurs in various types of tissues in developmental stages [20]. EMT not only participates in the formation and progression of tumors but also promotes invasion, migration, and other processes of malignant tumors and exerts a crucial effect on tumor relapse [21]. EMT involves changes of some molecules, such as epithelial cadherin (E-cadherin), neural cadherin (N-cadherin), vimentin, ZO-1, and A-SMA. Among them, E-cadherin and ZO-1 are downregulated, while vimentin, N-cadherin, and A-SMA are upregulated during EMT [22].

Although PVT1, YAP, and EMT have been intensively studied, the potential mechanism underlying their interaction is still unelucidated. In our study, we conducted an investigation into the interaction among PVT1, YAP, and EMT to evaluate their value in the diagnosis and therapeutics of GC.

## 2. Methods

### 2.1. TCGA Data Analysis

For analysis and exploration of the expression of lncRNA PVT1, data and information of GC and normal tissues were acquired from The Cancer Genome Atlas (TCGA), and the R software was used for statistical analysis with the relevant program package installing and loading. The expression difference of lncRNA PVT1 in 32 normal tissues and 381 gastric cancer tissues was analyzed, and the survival analysis was also conducted.

### 2.2. Clinical Specimen Collection

We collected 20 pairs of GC and adjacent nontumor tissues from GC patients who underwent treatment in the Affiliated Hospital of Qingdao University. All the tissue samples were identified by expert pathologists and were stored at −80°C. The study was approved by the Ethics Committee of the Affiliated Hospital of Qingdao University and strictly followed the standards of the Declaration of Helsinki. All the participants signed the informed consent documentation.

### 2.3. Cell Culture

GC (MKN-45, HGC-27) and normal human gastric mucosa (GES-1) cells were purchased from Wuhan Procell Life Technology Co., Ltd. (Wuhan, China). All the cells were cultured using 10% fetal bovine serum (FBS) (BI, South American Origin, Israel) containing 1% penicillin-streptomycin (PS, 100 mg/mL streptomycin and 100 units/mL penicillin) with Roswell Park Memorial Institute 1640 (RPMI 1640) (BI, Israel). Then the cells were subcultured every 2–3 days and incubated in a 5% CO incubator (Heracell™ VIOS 160i CO Incubator, Thermo Scientific™, USA) at 37°C.

### 2.4. Cell Transfection

PVT1 was knocked down using three PVT1-specific siRNAs (si-PVT1-608, si-PVT1-704, and si-PVT1-1748), and the negative control (NC) was developed using a nonsilencing siRNA (si-NC) (GenePharma, Shanghai, China).  Table 1 shows the sequences of siRNA. MKN-45 cells were inoculated in 6-well plates with 2 × 105 cells per well for 24 h before transfection and incubated overnight. We used Lipofectamine 3000 reagent (Invitrogen, Carlsbad, CA, USA) and Opti-MEM medium to transfect three siRNA-PVT1 and si-NC into the MKN-45 cells according to the specifications. RT-qPCR was used to determine the transfection efficacy. si-PVT1-1748 was selected for use in the following experiments because of its high transfection efficacy confirmed by RT-qPCR.

### 2.5. RNA Extraction and RT-qPCR

The 20 pairs of GC and adjacent nontumor tissues and cultured cells were used to extract the total RNA through TRIzol^®^ Reagent (TaKaRa Bio Inc., Otsu, Japan), and the concentration and purity of the extracted RNA were determined by the Multiskan™ FC Microplate Reader (Thermo Scientific™, USA). PrimeScript RT polymerase (TaKaRa Bio Inc., Otsu, Japan) was conducted to reversely transcribe total RNA (1 *μ*g) extracted from a 20 *μ*L reaction mixture to cDNA. SYBR Green PCR Master Mix (TaKaRa Bio Inc., Otsu, Japan) was used to conduct RT-qPCR to determine the expression level of RNA. GAPDH as an internal reference was used to detect the expression of lncRNA, and the reactions were performed on a Roche capillary-based LightCycler 2.0 system (Roche Diagnostics, Indianapolis, IN, USA). The PCR reactions were undertaken at 95°C for 30 s, 95°C for 5 s, and 60°C for 45 s for 40 cycles. The primers used for PCR were as follows: PVT1-forward: 5-TGCCCTGTTTGCTTCTCCTG-3, PVT1 reverse: 5′-TCTTTGACAGCCCCAAGCTG-3′; GAPDH-forward: 5-AACAGCCTCAAGATCATCAGCAA-3, GAPDH-reverse: 5′-GACTGTGGTCATGAGTCCTTCCA-3′. The 2^-ΔΔCt^ method was used to conduct comparative quantification, and every experiment was repeated three times.

### 2.6. Cell Counting Kit-8 (CCK-8) and Colony Formation Assay

GC (MKN-45, HGC-27) and normal human gastric mucosa (GES-1) cells (2 × 103 cells per well) were inoculated into 96-well plates and six wells were used for one assay group. Every well was supplied with 10 *μ*L CCK-8 solution (Meilunbio, Dalian, China) to incubate 48 h after transfection and then cultured for an extra 2 h at 37°C. The light absorbance was determined at 450 nm by the Multiskan™ FC Microplate Reader (Thermo Scientific™, USA). The ratio of the absorbance value of the experimental group to that in the control group was calculated, and then a cell viability chart was drawn.

Cells (2000 cells/well) were inoculated into 6-well plates and cultured routinely for 14 days. Then the cells were fixed in 10% formaldehyde for 15 min and stained with 0.1% crystal violet. The microscope (FLUOVIEW FV3000, Olympus, Japan) was used to count the number of cells. Colonies containing over 50 cells could be counted. Every experiment was also independently repeated three times.

### 2.7. Cell Migration and Invasion Assays

Both wound healing and Transwell assays were conducted to evaluate cell migration ability. Cells were inoculated into 6-well plates and cultured to 90% confluence. Subsequently, artificial scratches were made in the plates using a 20 *μ*l pipette tip. Cells were cultured in RPMI 1640 containing 2% FBS and imaged at 6 h, 12 h, 24 h, and 36 h through a microscope (FLUOVIEW FV3000, Olympus, Japan). All the cells (2.5 × 104) were cultured on a Transwell plate with 8 mm pores containing 200 *μ*l of serum-free RPMI 1640, supplemented with 10% FBS. The cells on the upper surface of the Transwell plate were removed using a cotton swab after a 24 h incubation and the cells on the lower surface were set for 30 min using 30% formaldehyde, which then were stained with 0.1% crystal violet and counted by a microscope (FLUOVIEW FV3000, Olympus, Japan).

We also conducted Transwell assays to evaluate cell invasion ability. Transwell chambers with 8 mm pores were precoated with Matrigel^®^ (BD, Franklin Lakes, NJ, USA). The cells (2.5 × 104) were cultured in a Transwell chamber containing 200 *μ*L of serum-free RPMI 1640 supplemented with 10% FBS. Cells on the lower surface were fixed, stained, and counted under a microscope. Each experiment was independently repeated three times.

### 2.8. Flow Cytometry Analysis

MKN-45 cells were applicable after 48 h following transfection, which were cleaved using 0.25% trypsin without EDTA and terminated with RPMI-1640 medium which contains 10% FBS. Then the cells were centrifuged at 1000 rpm for 5 min, washed twice with prechilled PBS, and resuspended in 1x binding buffer to regulate the cell density to 1 × 106 cells/ml. 5 *μ*L FITC-Annexin V and Propidium Iodide (PI) (Meilunbio, Dalian, China) were used to stain the cell suspension (100 *μ*L) for 15 min at 25°C in the dark. Apoptosis analysis was conducted by Apogee Flow Cytometers (Apogee Flow Systems, Hemel Hempstead, UK). Early apoptotic cells were positive for Annexin V and negative for PI (Q3), while late apoptotic cells were positive for both (Q2). The rate of apoptosis was determined by the sum of the proportions of early and late apoptotic cells (Q3 + Q2). Moreover, the flow cytometry software (FlowJo 10.0.7 system, Treestar, USA) was conducted to present and analyze the proportion of apoptotic cells in total. Each experiment was independently repeated three times.

### 2.9. Western Blotting

All the cell protein was extracted after transfection using RIPA buffer (Solarbio, Beijing, China) added with protease inhibitor cocktail and phosphatase inhibitor cocktail I (MCE, America) after washing the cells with PBS. A BCA kit (Elabscience Bio, Wuhan, China) was then used to quantify the concentration. 20 ug protein from each sample was extracted for electrophoresis on a 10% SDS-PAGE gel and it was transferred onto a polyvinylidene fluoride membrane (PVDF; Millipore, Billerica, MA, USA). Then, the membranes were blocked in 5% fat-free milk for 1 h at 25°C. Next the membranes were washed with TBST three times and incubated at 4°C overnight with primary antibodies, including anti-*β*-actin (4970T, 1 : 1000, CST), anti-YAP (14074T, 1 : 1000, CST), anti-p-YAP (13008T, 1 : 1000, CST), anti-vimentin (AB-70081, 1 : 1000, Elabscience), anti-E-cadherin (AB-53267,1 : 1000, Elabscience), and anti-zonula occludens-1 (ZO-1) (AB-18170, 1 : 1000, Elabscience). Furthermore, the membranes were washed three times with TBST, which were next incubated at 25°C for 1 h with respective horseradish peroxidase-labeled secondary antibodies. Finally, FUSION FX7 Spectra (Vilber, France) were conducted to acquire the protein bands. Each experiment was independently repeated three times.

### 2.10. Statistical Analysis

The statistical data was calculated and analyzed by GraphPad Prism 7 (GraphPad Software, Inc., USA) and SPSS 21.0 software (IBM Corp, Armonk, NY, USA). The results are presented as the mean ± standard deviation integrated with three independently replicated experiments. Data between two groups were compared and analyzed by Student's *t*-test. The survival rate analysis was performed by the Kaplan-Meier method, and the survival difference was analyzed by the log-rank test. *p* < 0.05 was considered statistically significant.

## 3. Results

### 3.1. PVT1 Is Upregulated in GC Tissues and Cells

**Figure 1:**
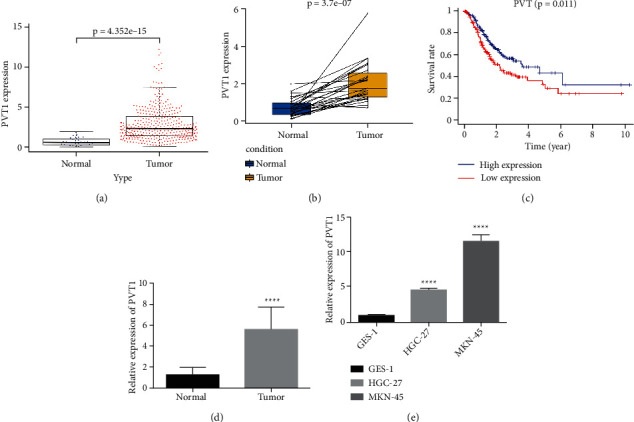
PVT1 is upregulated in GC tissues and cells. (a, b) The expression of PVT1 was analyzed using data from TCGA, and PVT1 was found to be upregulated in GC tissues. (c) Patient survival analyzed using data from TCGA, showing that the higher the expression of PVT1 is, the worse the prognosis is. (d) Expression of PVT1 in GC and adjacent normal tissues of 20 GC patients detected by RT-qPCR, showing that PVT1 was upregulated in GC tissues. (e) The expression of PVT1 detected by RT-qPCR in GES-1, HGC-27, and MKN-45 cell lines, showing that PVT1 was highest in MKN-45 cells.

The Cancer Genome Atlas (TCGA) datasets were used to identify the expression of lncRNA in GC using the R software. Compared with the nonneoplastic tissues, lncRNA PVT1 was remarkably upregulated (*P* < 0.0001) in GC tissues (Figures 1(a) and 1(b)), and the higher the expression level, the worse the prognosis (Figure 1(c)). Furthermore, PVT1 expression was downregulated in the adjacent nonneoplastic tissues compared with that in the GC tissues of 20 GC patients detected by RT-qPCR (Figure 1(d)). Lastly, our data revealed that PVT1 expression in the GC cells was higher than that in GES-1 cells and the highest expression was in MKN-45 cells (Figure 1(e)). These results suggest that PVT1 was upregulated in GC tissues and cells and negatively correlated with the survival of patients. Our results are consistent with those of previous research [23].

### 3.2. Knockdown of PVT1 Inhibits Tumor Cell Proliferation and Promotes Apoptosis

**Figure 2:**
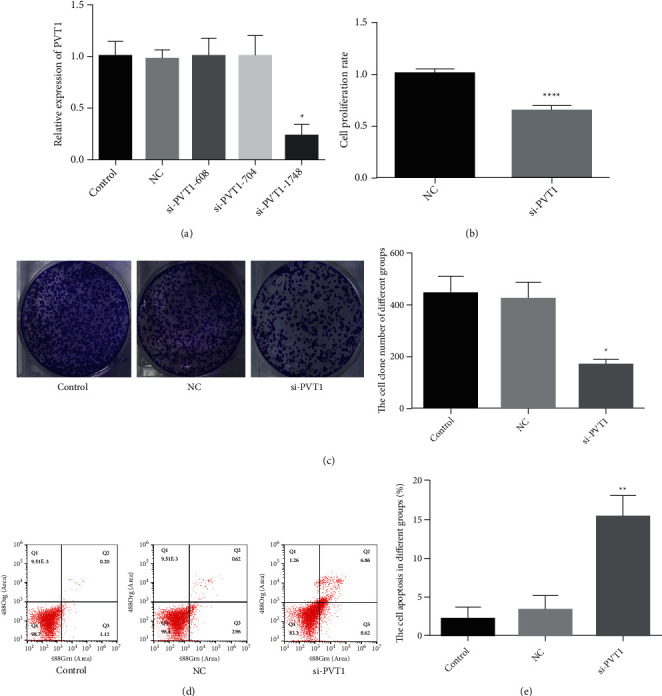
Knockdown of PVT1 inhibits tumor cell proliferation and promotes apoptosis of MKN-45 cells in vitro. (a) Transfection efficiency detected by RT-qPCR, showing that si-PVT1-1748 led to more effective inhibition compared to the other siRNAs. (b) The cell proliferation determined by CCK-8 assay, showing that knockdown of PVT1 inhibits tumor cell proliferation. (c) The cell proliferation of different groups analyzed by colony formation assay, showing that knockdown of PVT1 inhibits tumor cell proliferation. (d, e) The cell apoptotic rate of different groups determined by flow cytometry, showing that knockdown of PVT1 promotes tumor cell apoptosis.

To further explore and analyze the role of PVT1 on cell proliferation and apoptosis in MKN-45 cells, we transfected the MKN-45 cells with three PVT1-siRNAs (si-PVT1-608, si-PVT1-704, and si-PVT1-1748) and the negative control (si-NC). si-PVT1-1748 was selected for further experiments due to its higher efficacy of inhibition compared to others (Figure 2(a)). Knockdown of PVT1 significantly inhibited cell proliferation according to the result of the CCK-8 assay (Figure 2(b)). Furthermore, the colony formation assay suggested that knockdown of PVT1 caused an obvious decrease in the number of colonies formed (*P* < 0.05, Figure 2(c)). In addition, flow cytometric assays revealed that apoptosis was remarkably elevated in the si-PVT1-1748 group in comparison with the control and NC groups, and the percentage of apoptotic cells in the si-PVT1-1748 group increased significantly (*P* < 0.01, Figures 2(d) and 2(e)). Taken together, the proliferation of MKN-45 cells was inhibited and apoptosis was promoted after transfection with si-PVT1-1748 in vitro.

### 3.3. Knockdown of PVT1 Inhibits Tumor Cell Migration and Invasion

**Figure 3:**
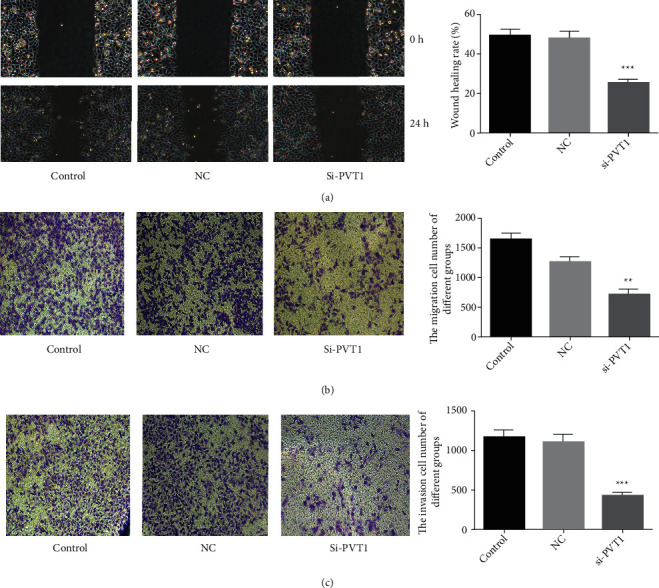
Knockdown of PVT1 inhibits tumor cell migration and invasion of MKN-45 cells in vitro. (a) The cell migration ability of different groups of cells studied by the wound-healing assay, showing that knockdown of PVT1 inhibits tumor cell migration. (b) The cell migration ability of different groups evaluated by the Transwell assay, showing that knockdown of PVT1 inhibits tumor cell migration. (c) The cell invasion ability of different groups analyzed by Transwell assay with Matrigel, showing that knockdown of PVT1 inhibits tumor cell invasion.

Cell migration and invasion lead to relapse or a poor prognosis in GC. Therefore, we examined whether PVT1 could regulate the migration and invasion ability of tumor cells. Knockdown of PVT1 led to inhibition of tumor cell migration in accordance with the result of the wound-healing assay (Figure 3(a)). Furthermore, the Transwell assay indicated that knockdown of PVT1 inhibited cell migration (Figure 3(b)) as well as invasion in MKN-45 cells (Figure 3(c)). The above results indicated that PVT1 knockdown resisted the migration and invasion of GC cells in vitro. In other words, PVT1 promoted tumor cell migration and invasion ability.

### 3.4. Knockdown of PVT1 Inhibits the Expression of YAP and EMT-Related Proteins

**Figure 4:**
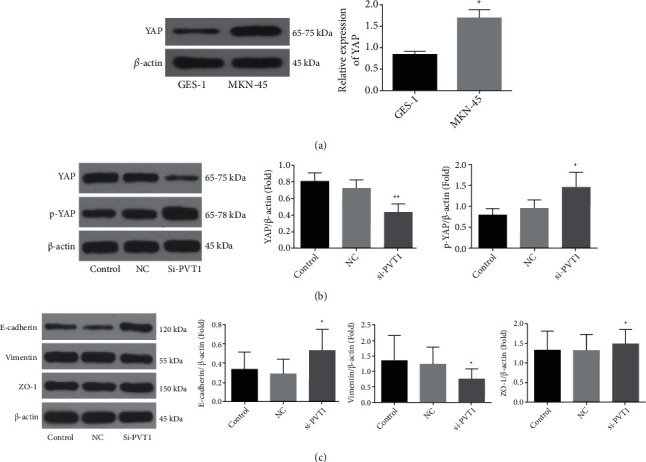
Knockdown of PVT1 inhibits the expression of YAP and EMT proteins.

Previous studies have suggested that YAP serves as an oncogene in many tumors [24, 25]. Western blotting indicated that YAP was upregulated in GC cells (Figure 4(a)). We transfected MKN-45 cells with si-PVT1-1748 and negative control (si-NC) and discovered that knockdown of PVT1 inhibited the expression of YAP (Figure 4(b)), which indicated that the expressions of PVT1 and YAP were positively associated. In addition, knockdown of PVT1 increased the expression level of p-YAP but decreased the expression level of YAP. When YAP binds to a phosphorylated ser127, it will accumulate in the cytoplasm and cannot enter into the nucleus, losing its function as a transcriptional coactivator and exerting a pleiotropic effect on tumor growth and suppression of apoptosis. These results indicated that knockdown of PVT1 could suppress a series of malignant biological behaviors of GC cells including proliferation, invasion, and migration and enhance cell apoptosis by inducing the phosphorylation of YAP.

EMT plays a crucial role in the formation and development of multiple cancers, which is also a very important factor affecting the metastasis and relapse of various tumors. EMT is accompanied by increased expression of vimentin and decreased expression of E-cadherin and ZO-1. Western blotting analysis showed that knockdown of PVT1 causes downregulation of vimentin as well as upregulation of E-cadherin and ZO-1, inhibiting the EMT process (Figure 4(c)).

Western blotting on the expression of different groups of cells, showing that knockdown of PVT1 inhibits the expression of YAP and increases the expression of p-YAP. Meanwhile, knockdown of PVT causes upregulation of E-cadherin and ZO-1 and downregulation of vimentin.

### 3.5. The Relationship among PVT1, YAP, and EMT in GC

**Figure 5:**
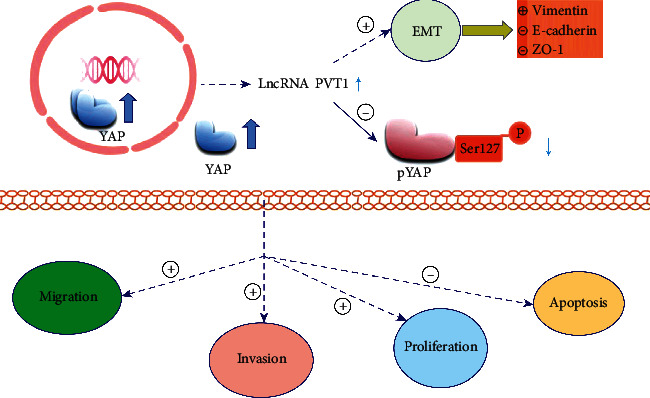
The relationship among PVT1, YAP, and EMT in GC. PVT1 and YAP were both upregulated in GC tissues. In GC, PVT1 promotes the expression of YAP, inhibits the expression of p-YAP, promotes tumor cell proliferation, tumor cell migration, and invasion, and inhibits cell apoptosis. The increased expression of PVT1 promotes EMT.

As shown in Figure 5, PVT1 and YAP were both upregulated in GC tissues. In GC, PVT1 might promote the expression level of and while inhibit the expression level of p-YAP. Therefore, PVT1 promotes tumor cell malignant biological behaviors including proliferation, migration, and invasion and inhibits cell apoptosis. Meanwhile, the increased expression of PVT1 promotes EMT. Knockdown of PVT1 inhibits the expression of YAP, increases the expression of p-YAP, and inhibits the EMT process, causing increased expressions of E-cadherin and ZO-1 and decreased expression of vimentin. In summary, the interaction among PVT1, YAP, and EMT is crucial in the progression of GC.

## 4. Discussion

GC as one of the malignancies causes increased morbidity and dismal mortality worldwide [26, 27]. Previous studies have elucidated that the formation and progression of tumors involved a variety of molecular changes, such as abnormal protein or gene expression [28]. Despite advances in treatment, the prognosis of GC is dismal due to tumor recurrence and metastasis [5]. Many lines of evidence show that lncRNAs may serve as oncogenes or tumor suppressor genes to modulate gene expression and affect cell proliferation, migration, invasion, apoptosis, and other pathological processes [29], including the progression of various human diseases, particularly cancers [7]. The aberrant expression of lncRNAs is correlated with the formation and development of GC, which can be considered as molecular biomarkers and targets for diagnosis and therapeutics [30]. For example, the expression of H19 is upregulated in GC compared with normal tissues and promotes the development of GC through miR-223p-mediated upregulation of Snail1 [31]. It has been reported that lncRNA MALAT1 acting as an oncogenic gene in GC might be a novel biomarker and target for the diagnosis of GC [32]. Multiple studies have clarified that the expression of PVT1 is lower in normal tissues but higher in various malignant cancer tissues and cancer cell lines, promoting tumor progression [13, 14, 33–36]. Thus, we conducted experiments to further explore the potential effect of PVT1 on the proliferation, apoptosis, invasion, and migration of GC cells and investigate its possible mechanism.

In our study, we first analyzed information from 32 normal tissues and 381 GC tissues in the TGCA database and discovered that PVT1 was remarkably elevated in GC tissues compared with normal tissues. In the meantime, we found that PVT1 expression was negatively correlated with the patient survival rate. To verify this result, gastric mucosa cells (GES-1) and GC cells (HGC-27, MKN-45) were selected as subjects. As a result, the expression of PVT1 in HGC-27 and MKN-45 was significantly higher than that in GES-1. Subsequently, we performed functional experiments after silencing PVT1 with siRNA, which indicated that knockdown of PVT1 significantly resisted the malignant biological behaviors of MKN-45 and promoted its apoptosis. In summary, our study elucidated that PVT1 could act as an oncogenic gene and might be a valuable target for the therapeutics of GC.

The previous study has shown that YAP expression was elevated in tumors, including GC [37]. With the deepening of research, increasing lines of evidence show that proteins interact with lncRNA in the occurrence and progression of tumors [38], while the relationship between PVT1 and YAP in GC has not been elucidated. Therefore, we explored the relationship between PVT1 and YAP through experiments. In our study, YAP expression was positively correlated with PVT1. Knockdown of PVT1 led to the downregulation of YAP expression and upregulation of p-YAP. When YAP is phosphorylated, it accumulates in the cytoplasm, preventing it from entering into the nucleus and losing its function as a transcriptional coactivator. It was revealed that PVT1 knockdown suppressed the proliferation, migration, and invasion ability of GC cells and facilitated apoptosis in vitro by promoting the phosphorylation of YAP.

Increasing lines of evidence have indicated that EMT plays a vital role in tumor progression. EMT is a cellular programmer that is known to be crucial for embryogenesis, wound-healing, and malignant progression [21, 39]. EMT involves molecules of E-cadherin, N-cadherin, vimentin, ZO-1, and A-SMA [40]. One study has shown that the expressions of molecular markers of EMT are significantly upregulated in bladder cancer and promoted malignant biological behaviors of bladder cancer cells [41]. In our study, the expressions of E-cadherin and ZO-1 were upregulated after the knockdown of PVT1, while the expression of vimentin was downregulated, indicating that knockdown of PVT1 inhibits EMT. These data further suggest that inhibition of the EMT signaling pathway can inhibit the progression of GC. We have confirmed that the interaction among PVT1, YAP, and EMT is crucial in the progression of GC.

However, the number of GC tissue samples for histopathological validation is limited. Further research is needed to confirm the results in our study including in vivo experiments, preclinical studies, and clinical trials, and detailed mechanisms among PVT1, YAP, and EMT warrant further exploration. In addition, the subsequent study could also explore the role of the lncRNA-miRNA-mRNA network in the carcinogenesis mechanism of GC.

In summary, knockdown of PVT1 inhibits malignant biological behaviors of GC cells and facilitates apoptosis by promoting the phosphorylation of YAP. PVT1 also regulates the epithelial-mesenchymal transition process. Our study provides a rationale for knockdown of PVT1 in the treatment of GC. Therefore, PVT1 and YAP may be novel biomarkers and valuable targets for the diagnosis, treatment, and prognosis of GC.

## Figures and Tables

**Table 1:** Sequences of siRNA.

Gene	Sequences (5′–3′)
PVT1-homo-608	CCUGUUACACCUGGGAUUUTT
	AAAUCCCAGGUGUAACAGGTT
PVT1-homo-1748	GCUUCAACCCAUUACGAUUTT
	AAUCGUAAUGGGUUGAAGCTT
PVT1-homo-704	GGGAAUCACUACUGACCUUTT
	AAGGUCAGUAGUGAUUCCCTT
Human GAPDH	CACUCAAGAUUGUCAGCAATT
	UUGCUGACAAUCUUGAGUGAG
Negative control	UUCUCCGAACGUGUCAGCUTT
	AGCUGACACGUUCGGAGAATT
Negative control FAM	UUCUCCGAACGUGUCACGUTT
	ACGUGACACGUUCGGAGAATT

GAPDH is positive control, and FAM is fluorescence labeling.

## Data Availability

The datasets (TCGA) for this study can be found in The Cancer Genome Atlas (https://www.cancer.gov/about-nci/organization/ccg/research/structural-genomics/tcga). All other data are available upon reasonable request by writing to the corresponding author.
